# Dominant-Negative Attenuation of cAMP-Selective Phosphodiesterase PDE4D Action Affects Learning and Behavior

**DOI:** 10.3390/ijms21165704

**Published:** 2020-08-09

**Authors:** Graeme B. Bolger, Lisa High Mitchell Smoot, Thomas van Groen

**Affiliations:** 1 Department of Medicine and Department of Pharmacology, University of Alabama at Birmingham, Birmingham, AL 35294-3300, USA; 2 BZI Pharma LLC, 1500 1st Ave N., Unit 36, Birmingham, AL 35203-1872, USA; 3 Department of Medicine, University of Alabama at Birmingham, Birmingham, AL 35294, USA; lsmoot@uab.edu; 4 Department of Cell, Developmental and Integrative Biology, University of Alabama at Birmingham, Birmingham, AL 35294, USA; vangroen@uab.edu

**Keywords:** learning, memory, PKA, CREB, PDE4, PDE4D5, RACK1, β-arrestin2, acrodysostosis

## Abstract

PDE4 cyclic nucleotide phosphodiesterases reduce 3′, 5′ cAMP levels in the CNS and thereby regulate PKA activity and the phosphorylation of CREB, fundamental to depression, cognition, and learning and memory. The PDE4 isoform PDE4D5 interacts with the signaling proteins β-arrestin2 and RACK1, regulators of β_2_-adrenergic and other signal transduction pathways. Mutations in *PDE4D* in humans predispose to acrodysostosis, associated with cognitive and behavioral deficits. To target PDE4D5, we developed mice that express a PDE4D5-D556A dominant-negative transgene in the brain. Male transgenic mice demonstrated significant deficits in hippocampus-dependent spatial learning, as assayed in the Morris water maze. In contrast, associative learning, as assayed in a fear conditioning assay, appeared to be unaffected. Male transgenic mice showed augmented activity in prolonged (2 h) open field testing, while female transgenic mice showed reduced activity in the same assay. Transgenic mice showed no demonstrable abnormalities in prepulse inhibition. There was also no detectable difference in anxiety-like behavior, as measured in the elevated plus-maze. These data support the use of a dominant-negative approach to the study of PDE4D5 function in the CNS and specifically in learning and memory.

## 1. Introduction

Highly selective inhibitors of the PDE4, 3′, 5′-cAMP-specific phosphodiesterases have proven cognition-enhancing and antidepressant properties in humans [1,2,3,4,5,6,7,8,9,10,11,12] and rodents [13,14,15,16,17,18,19,20,21,22,23,24,25,26,27,28,29,30,31,32,33,34,35,36,37,38,39,40,41,42,43,44,45,46,47,48]. PDE4-selective inhibitors are exploited therapeutically for their anti-inflammatory, immunomodulatory and smooth-muscle relaxant activities (for reviews, see [49,50,51]). At present, three PDE4-selective inhibitors, roflumilast, apremilast, and crisaborole, are in clinical use for a wide range of respiratory and inflammatory disorders [52,53,54]. A PDE3/4-selective inhibitor, ensifentrine (RPL554), is in mid-stage clinical trials for chronic obstructive pulmonary disease [55,56]. A number of PDE4-selective inhibitors are under development specifically for CNS indications, including depression and as enhancers of learning and memory [9,10,11,12,45,46,47,48,57,58,59,60,61,62,63].

PDE4-selective inhibitors inhibit the enzymatic hydrolysis of cAMP and thereby increase its levels in cells. One of their major effects is to activate cAMP-dependent protein kinase (PKA) and thereby stimulate the phosphorylation of its physiological substrates, including the cyclic nucleotide response element-binding protein (CREB). Activation of CREB is essential for numerous CNS functions, notably learning and memory (for reviews, see [64,65,66]) and depression [67]. Additional targets of cAMP in the CNS include the exchange protein directly activated by cAMP (EPAC), cyclic-nucleotide-gated ion channels, and popeye-domain-containing proteins [68,69,70,71]. There are over 20 PDE4 isoforms, which are encoded by 4 genes in mammals (in humans, *PDE4A*, *PDE4B*, *PDE4C*, and *PDE4D*), each of which produces a range of isoforms by the use of alternative promoters and alternative mRNA splicing specific for each isoform [72,73]. Each isoform has a distinct tissue-specific pattern of mRNA and protein expression, including significant regional expression differences in the CNS, suggesting that each has a distinct biological function [74,75,76,77,78,79,80,81,82,83,84]. All PDE4-selective inhibitors that have been synthesized to date interact with the catalytic sites of the PDE4 enzymes and therefore act, to a considerable extent, as competitive inhibitors of cAMP hydrolysis [49,85,86,87]. The catalytic sites of the various PDE4 isoforms are extremely similar, posing a challenge to the development of truly isoform-selective PDE4 inhibitors [86].

**Figure 1:**
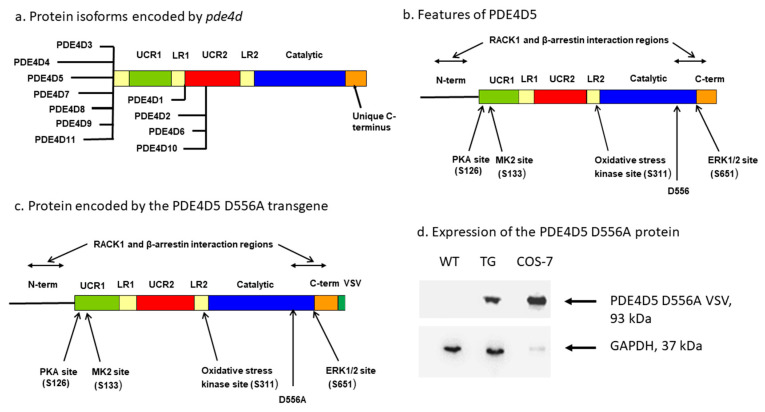
(**a**) Proteins encoded by the human *PDE4D* gene [73,88,96,97,98,99,100]. The long isoforms PDE4D3, PDE4D4, PDE4D5, PDE4D7, PDE4D8, PDE4D9, and PDE4D11 all contain UCR1, UCR2, and catalytic domains, separated by the unstructured LR1 and LR2 regions. The short isoform PDE4D1 contains the catalytic domain and UCR2, while the super-short PDE4D2, PDE4D6, and PDE4D10 isoforms contain the catalytic region and a portion of UCR2. UCR1 and UCR2 mediate dimerization of the long PDE4D isoforms. Each isoform also contains isoform-specific unique regions at its amino-terminus (heavy black lines). The carboxyl-terminal region (C-terminus) is also shown, which is common to all PDE4D isoforms but which differs from the carboxyl-terminal regions of PDE4A, PDE4B, and PDE4C isoforms. (**b**) Features of PDE4D5. The 88-amino-acid unique amino-terminal region (N-term) of PDE4D5 is indicated (heavy black line), along with the C-terminus (C-term) and regions required for its interaction with RACK1 and β-arrestin2. The PKA site and MK2 site are also shown, both located within UCR1, the oxidative stress kinase site located within LR2, the ERK1/2 site located in the catalytic region, and D556, the amino acid mutated in this study. (**c**) Protein encoded by the PDE4D5 D556A transgene. Features as in b, but with the D556A mutation and the VSV epitope at the carboxyl-terminus. (**d**) Immunoblotting of whole-brain extracts (10 μg protein/lane) from wild-type (WT) and transgenic (TG) mice. Extract from COS-7 cells (1 ug protein/lane) transfected to express PDE4D5-D556A-VSV was run as a size standard (COS-7). Immunoblotting with anti-VSV detected a 93-kDa protein (top). Immunoblotting with anti-GAPDH, serving as a loading control, detected a 37-kDa protein (bottom).

One of the best-studied PDE4 isoforms is PDE4D5, which is 1 of 11 isoforms encoded by the mouse *Pde4d* gene and which is highly conserved among mammals (Figure 1a,b, [88]). Each PDE4D isoform is expressed off a different promoter and has a unique expression pattern in the CNS [74,76,77,78,82,89,90,91,92,93,94,95]. For example, in situ studies using riboprobes have shown that each of the PDE4D1, -2, -3, -4, and -5 mRNAs (Figure 1a) have a distinct distribution pattern in the brain, correlating in some cases with specific brain pathways. PDE4D2 mRNA transcripts are expressed in the dorsal and median raphe nuclei, probably located in serotonergic cells. PDE4D1 and PDE4D2 mRNA splice forms are both expressed in the area postrema, whereas the cellular distribution of PDE4D4 and PDE4D5 shows a complementary pattern [77]. Other studies have shown that PDE4D isoforms are localized to neurons in the nodose ganglion and in many structures of the medulla, including neurons of the nucleus tractus solitarius and locus coeruleus [89]. Collectively, these data suggest that each isoform has a unique partially non-overlapping role; however, this needs to be confirmed by functional studies of each of these isoforms.

PDE4D5 interacts selectively and at high avidity with several signaling proteins, most notably RACK1 [101,102,103,104,105,106,107], β-arrestin2 [103,108,109,110,111,112,113,114], and also several elements of MAPK signaling, notably ERK1/2 [115,116,117] and MK2 [118,119]. RACK1 and β-arrestin2 both interact much more avidly with PDE4D5 than with any other PDE4D isoform [101,110]. PDE4D5 is also the target of several additional kinases, most notably PKA [120,121,122,123] and oxidative-stress kinases [124] (Figure 1b). Collectively, the phosphorylation state of PDE4D5 that is produced by the cumulative effects of these kinases alters the enzymatic activity of the kinase (velocity), its affinity for its substrate, cAMP, and various PDE4-selective inhibitors, its interactions with its protein partners, and its intracellular localization.

To expand our knowledge of the functional roles of various PDE4 isoforms in the CNS, and as a stimulus to drug development, we adopted a dominant-negative approach. We and our collaborators utilized this approach initially to study the functions of PDE4D5 in cell-based assays [103,108,109,111]. This dominant-negative mutation is in a single amino acid (D556A in PDE4D5, conserved in all PDE4 isoforms) in a specific metal-ion-binding site in the catalytic region of PDE4D5 that has been identified by X-ray crystallographic analysis as being essential for catalysis [125,126]. Mutation of this amino acid to alanine dramatically reduces (<99% reduction) the enzyme’s catalytic activity [103,108,109]. When PDE4D5-D556A is overexpressed in cells, this mutant does not detectably change total PDE4D enzymatic activity but produces an equilibrium displacement of endogenous PDE4D5 from its protein partners, most notably RACK1 and β-arrestin2, or affects the ability of the PDE4 isoform to homodimerize [107,127,128,129,130,131], and therefore disrupts the cellular functions of PDE4D5 in a dominant-negative fashion [103,108,109,111,132]. The cellular effects of PDE4D5-D556A have been studied most extensively in cells exhibiting the action of the β_2_-adrenergic receptor (β_2_AR; [103,108,109,111]). In these experiments, PDE4D5-D556A-mediated displacement of β-arrestin2 from wild-type PDE4D5 prevented β-arrestin2 from recruiting wild-type PDE4D5 to the β_2_AR, thereby blocking cAMP-mediated downregulation of the β_2_AR by PKA [108,109]. This blocked the β-arrestin2-mediated switch of the β_2_AR from G_s_ to G_i_ and thereby activated the ERK1/2 kinase pathway [103,109,111].

Recently, we extended our dominant-negative approach to the functional study of PDE4 isoforms in the CNS [133]. Our first study focused on an important PDE4B isoform, specifically PDE4B1. For this work, we developed mice that expressed a dominant-negative mutant, PDE4B1-D564A (corresponding to the D556A mutation in PDE4D5), in the murine forebrain and hippocampus. This PDE4B1-D564A-encoding transgene markedly affected behavior, as measured in open-field testing, and produced significant alterations in the phosphorylation of critical substrates and in hippocampal synaptic plasticity [133]. This and similar dominant-negative strategies [134,135,136] expand on other genetic approaches that have been used to study PDE4 function in the CNS, such as gene knockouts [27,137,138,139,140,141,142,143] and lentiviral siRNA [142,144,145,146,147], but are potentially more isoform-selective.

Cell-based assays and animal studies have demonstrated a plausible functional role for PDE4D5 in numerous cellular functions, including β_2_-adrenergic signaling [108,109,111,112,148,149], airway smooth muscle function [150,151,152], cell spreading and motility in culture [153], and immunity/inflammation [154]. However, given the significant CNS actions of PDE4-selective inhibitors, we were particularly interested in determining the precise function(s) of PDE4D5 in the CNS. Therefore, we aimed to determine whether the PDE4D5-D556A mutant, when expressed as a transgene in the brain, affected behavior. Given the potential CNS uses of PDE4-selective inhibitors, and the potential correlations between depressive disorders and cognitive and neurophysiological symptoms [58,59], we were particularly interested in testing the effect of PDE4D5-D556A on cognition, activity, learning and memory, and on measures of anxiety or depression.

## 2. Results

### 2.1. Mice Expressing a PDE4D5-D556A Transgene in the CNS

Our transgenic mice expressed PDE4D5-D556A (Figure 1c) under the control of the α-calmodulin kinase II (αCaMKII) promoter [155,156,157]. They were generated specifically for this paper using techniques we described previously [133]. During breeding, the PDE4D5-D556A transgene was inherited at the expected Mendelian frequency, showing that the transgene did not produce toxicity or affect fetal viability. PDE4D5-D556A transgenic mice were grossly indistinguishable from wild-type littermates and commercially available C57BL/6J mice. They grew at a normal rate and attained a normal adult size. Immunoblotting detected the expression of PDE4D5-D556A in transgenic but not wild-type, mice (Figure 1d).

### 2.2. Basic Neurologic Functions in PDE4D5-D556A Transgenic Mice

We assessed basic aspects of PDE4D5-D556A mice using the SHIRPA protocol [158,159]. This protocol tested measurements of physical characteristics, sensorimotor reflexes, general behavior, motor responses, grip strength, and included the Rotarod test and the wire suspension test, as described [133]. Wild-type and PDE4D5-D556A transgenic mice littermates were indistinguishable in these assays.

### 2.3. Effects of the PDE4D5-D556A Transgene on Activity in a Novel Open Field

**Figure 2:**
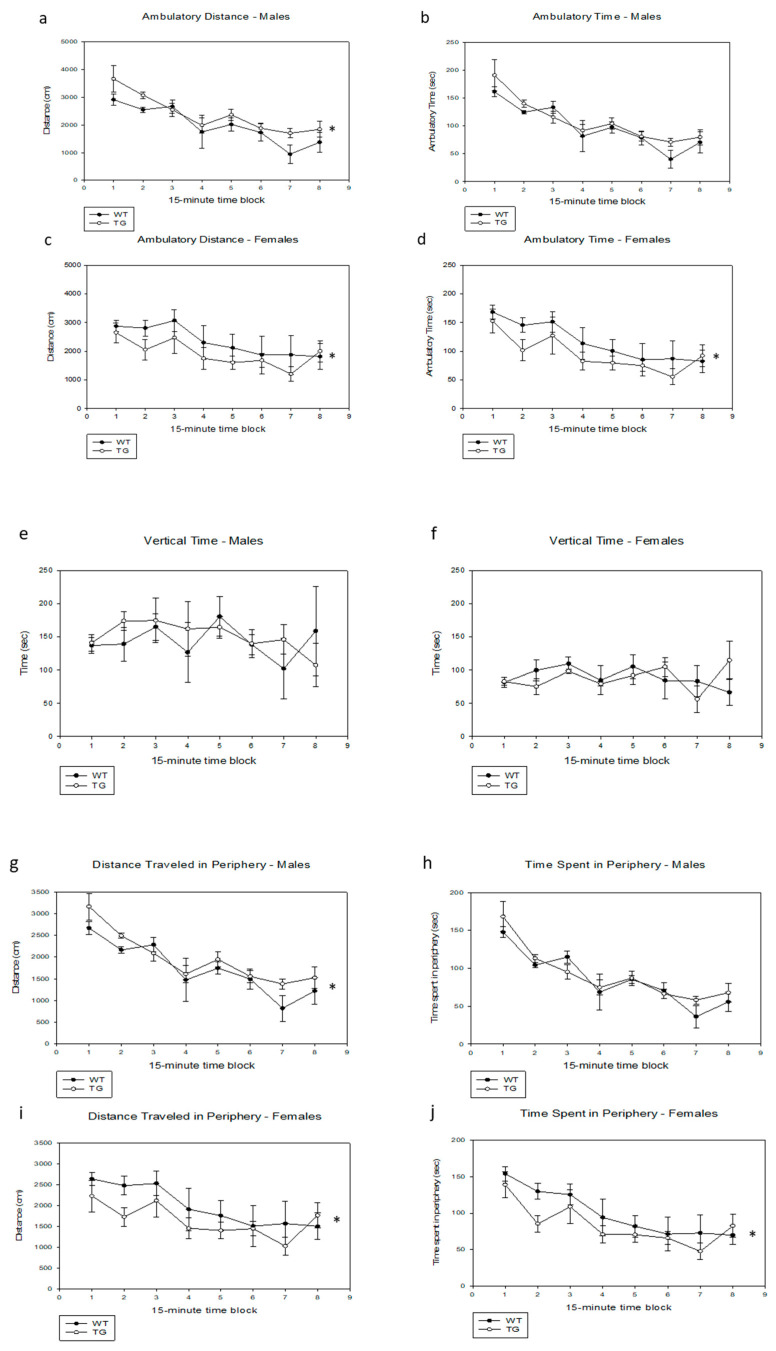
Activity of PDE4D5-D556A transgenic mice in an open field. Testing was performed for 2 h and activity analyzed for each 15-min block. Data are means ± SE; asterisks (*) indicate data from the transgenic mice that were statistically different over the 2-h testing period from that obtained from wild-type mice. (**a**) Ambulatory distance, males (**b**) Ambulatory time, males (**c**) Ambulatory distance, females (**d**) Ambulatory time, females (**e**) Vertical time, males (**f**) Vertical time, females (**g**) Distance traveled in periphery, males (**h**) Time spent in periphery, males (**i**) Distance traveled in periphery, females (**j**) Time spent in periphery, females.

We noted no significant difference between wild-type and PDE4D5-D556A transgenic littermates after only 10 min of observation in an open field. However, over a 2-h observation period, we noted statistically significant differences in both males and females (Figure 2). PDE4D5-D556A transgenic males, compared to wild-type males, had increased total activity (total ambulatory distance, Figure 2a, TG vs. WT, *p* = 0.008, F(1,7) = 13.29; for all male comparisons, *n* = 5 WT, *n* = 5 TG), and total ambulatory time (Figure 2b, *p* = 0.09, F(1,7) = 3.83). Females showed the opposite effect (Figure 2c,d, total ambulatory distance, TG vs. WT, *p* = 0.007, F(1,7) = 13.87; total ambulatory time, *p* = 0.007, F(1,7) = 13.52; for all female comparisons, *n* = 5 WT, *n* = 5 TG). Ambulatory distance for the total 2-h period was 2380 ± 54 cm for transgenic males compared to 1992 ± 208 cm for wild-type males, and 1928 ± 293 cm for transgenic females compared to 2342 ± 403 cm for wild-type females (means ± SEM). We saw no detectable effect on vertical time (data for males, Figure 2e, TG vs. WT, *p* = 0.5, F(1,7) = 0.48, and for females, Figure 2f, TG vs. WT, *p* = 0.9, F(1,7) = 0.28). The effect of the PDE4D5-D556A transgene in males appeared robust, in that transgenic males also showed increased activity in the periphery of the field (ambulatory distance in the periphery, Figure 2g, TG vs. WT, *p* = 0.028, F(1,7) = 7.60) although ambulatory time in the periphery was not different (Figure 2h, TG vs. WT, *p* = 0.26, F(1,7) = 1.50). The reverse effect seen in females also appeared to be robust (Figure 2i,j, ambulatory distance in periphery, TG vs. WT, *p* = 0.017, F(1,7) = 9.68, and ambulatory time in periphery, *p* = 0.028, F(1,7) = 7.63).

As is typical for this assay, activity declined over the 2-h time course of the experiment. This effect was similar between PDE4D5-D556A transgenic and wild-type males (first vs. second hour, TG vs. WT: total ambulatory distance, *p* = 0.8, F(1,7) = 0.054; total ambulatory time, *p* = 0.02, F(1,7) = 8.50; distance in periphery, *p* = 0.7, F(1,7) = 0.14; time in periphery, *p* = 0.7, F(1,7) = 0.13). It was also similar between PDE4D5-D556A transgenic and wild-type females (first vs. second hour, TG vs. WT: total ambulatory distance, *p* = 0.5, F(1,7) = 0.41; total ambulatory time, *p* = 0.4, F(1,7) = 0.69; distance in periphery, *p* = 0.3, F(1.7) = 1.13; time in periphery, *p* = 0.3, F(1,7) = 1.28).

### 2.4. Lack of Effect of the PDE4D5-D556A Transgene on Acoustic Startle and Prepulse Inhibition (PPI)

**Figure 3:**
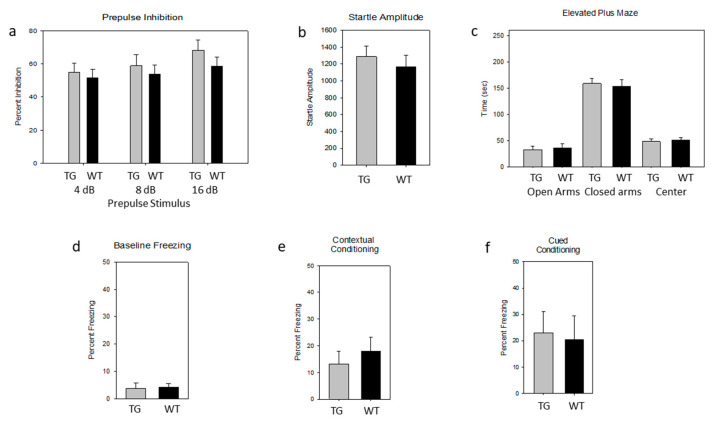
PDE4D5-D556A transgenic mice show no clear differences in prepulse inhibition, anxiety, or fear conditioning. Black bars: Wild-type (WT). Grey bars: PDE4D5-D556A (TG). Sexes are pooled. Data are means ± SE. (**a**) Prepulse inhibition, percent inhibition with progressive stimulus (percent inhibition at prepulses of 4, 8, and 16 dB over background). (**b**) Prepulse inhibition, amplitude of startle response. (**c**) Elevated plus maze, time in each arm. (**d**) Fear conditioning, baseline freezing. (**e**) Fear conditioning, contextual conditioning. (**f**) Fear conditioning, cued conditioning.

PDE4-selective inhibitors have been investigated as a potential therapy for schizophrenia and major affective disorders [28,83]. Of the various PDE4 isoforms, those encoded by *PDE4B* have been most closely associated with schizophrenia and major affective disorders and have been shown to interact directly with DISC1, a protein implicated in neurodevelopment, nuclear functions, and susceptibility to schizophrenia in humans [160,161,162,163,164]. Mice with mutations in *Disc1* have a diverse behavioral phenotype, including alterations in PPI [83,165,166]. However, in a recent study, we were unable to detect any effect of the PDE4B1-D564A transgene on PPI [133]. Given these considerations, we were motivated to determine any potential effect of the PDE4D5-D556A transgene on PPI (Figure 3a,b). We were unable to detect any difference in PPI in PDE4D5-D556A transgenic mice (Figure 3a; inhibition (%) at prepulses of 4, 8, and 16 dB above background, TG vs. WT, sexes pooled: *p* = 0.64, 0.56 and 0.26, respectively, F(1,18) = 0.22, 0.35 and 1.34, respectively; for males: *p* = 0.18, 0.08 and 0.016, respectively, F(1,8) = 2.16, 3.80, 9.26, respectively; for females: *p* = 0.55, 0.39, 0.63, respectively, F(1,8) = 0.38, 0.82, and 0.26, respectively). There was no detectable difference in the startle amplitude (Figure 3b; sexes pooled: *p* = 0.52, F(1,18) = 0.43; *n* = 10 WT, *n* = 12 TG; for males: *p* = 0.12, F(1,8) = 2.99; *n* = 5 WT, *n* = 5 TG; for females: *p* = 0.4, F(1,8) = 0.77, *n* = 5 WT, *n*= 5 TG). The overall magnitude of acoustic startle and PPI in our mice was also typical of that seen in the C57BL6/J background [167].

### 2.5. Lack of an Effect of the PDE4D5-D556A Transgene on Tests of Anxiety

We used the elevated plus maze to assess for a potential anxiogenic effect of the PDE4D5-D556A transgene. We tested both the time spent in the open arms (Figure 3c) and exploration attempts. PDE4D5-D556A transgenic mice, compared to their wild-type littermates, spent indistinguishable amounts of time in each arm (TG vs. WT, sexes pooled: *p* = 0.54, F(9,1) = 0.40 for closed arms; *p* = 0.69, F(9,1) = 0.164 open arms; *p* = 0.58, F(9,1) = 0.32 for center, *n* = 10 WT, *n* = 10 TG), with no detectable difference by sex (females: *p* = 0.87, F(4,1) = 0.0289 for closed arms; *p* = 0.82, F(4,1) = 0.055 for open arms; *p* = 0.98, F(4,1) = 0.008 for center, *n*= 5 WT, *n*= 5 TG; males: *p* = 0.49, F(4,1) = 0.56 for closed arms; *p* = 0.78, F(4,1) = 0.09 for open arms; *p* = 0.46, F(4,1) = 0.66 for center, *n* = 5 WT, *n* = 5 TG).

### 2.6. Lack of an Effect of the PDE4D5-D556A Transgene on Fear-Associated Conditioning

PDE4-selective inhibitors have potential therapeutic value in disorders of human learning and memory (see background). A defect in fear-associated conditioning is a hallmark of mice deficient in CREB [65,66,168,169,170,171,172], a PKA substrate that is regulated in part by PDEs [49,50]. Therefore, we tested PDE4D5-D556A transgenic and wild-type mice in a typical fear-associated conditioning protocol, with measurement of the baseline freezing (Figure 3d) and both context-dependent and cue-dependent conditioning (Figure 3e,f). We found no detectable difference between PDE4D5-D556A transgenic and wild-type mice in baseline freezing (i.e., prior to the presentation of the cue; Figure 3d; percent freezing, TG vs. WT, sexes pooled: *p* = 0.85, F(8,1) = 0.04, *n* = 9 WT, *n* = 9 TG; for males: *p* = 0.29, F(3,1) = 1.68, *n* = 5 WT, *n* = 4 TG; for females: *p* = 0.97, F(3,1) = 0.001, *n* = 4 WT, *n* = 5 TG). We also detected no difference in freezing on exposure to a novel context after contextual conditioning (Figure 3e; percent freezing, sexes pooled: *p* = 0.40, F(9,1) = 0.78, *n* = 10 WT, *n* = 10 TG; for males: *p* = 0.56, F(4,1) = 0.40; *n* = 5 WT, *n* = 5 TG; for females: *p* = 0.59, F(4,1) = 0.33, *n* = 5 WT, *n* = 5 TG). We also detected no difference in freezing after cued conditioning (Figure 3f; percent freezing with stimulus, sexes pooled: *p* = 0.80, F(9,1) = 0.07; *n* = 10 WT, *n* = 10 TG; for males: *p* = 0.74, F(4,1) = 0.12, *n* = 5 WT, *n* = 5 TG; for females: *p* = 0.46, F(4,1) = 0.66, *n* = 5 WT, N = 5 TG).

### 2.7. Effects of the PDE4D5-D566A Transgene on Spatial Learning

**Figure 4:**
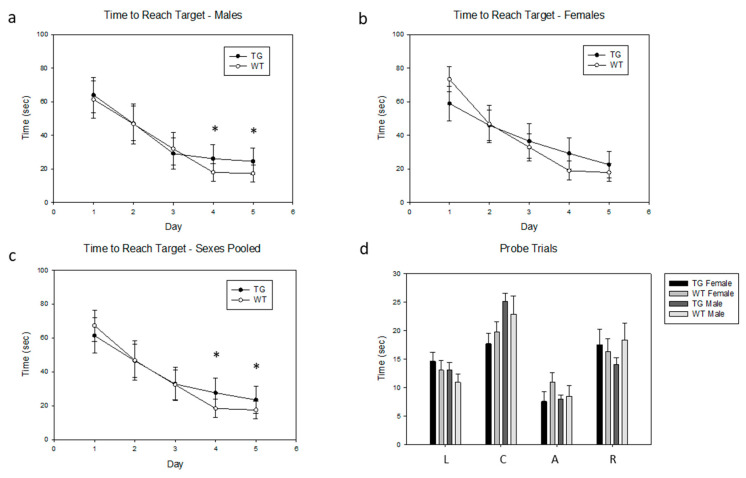
PDE4D5-D556A male mice show increased latency (time to target) in the Morris water maze. Data are means ± SE; asterisks (*) indicate data from the transgenic mice that were statistically different over days 4 and 5 from that obtained from wild-type mice. (**a**) Time to reach target during the training phase, males. (**b**) Time to reach target during the training phase, females. (**c**) Time to reach target during the training phase, sexes pooled. (**d**) Probe trials: time spent in each quadrant for each genotype and sex. L: left quadrant; C: contralateral quadrant; A: quadrant that previously contained the target; R: right quadrant.

Spatial learning is dependent, at least in part, on hippocampal function and is defective in mice with mutations in CREB [65,66,168,169,170,171,172] and mutations in other elements of cAMP signaling pathways, including adenylyl cyclase [173] and Epac [174,175]. Therefore, we assessed the effects of the PDE4D5-D556A transgene on spatial learning, using the Morris water maze [176]. Male PDE4D5-D556A transgenic mice demonstrated significant impairment in spatial learning, in that their time to reach the target in the water maze was significantly longer than for wild-type mice (Figure 4a). This effect was detectable on days 4 and 5 of the typical 5-day training protocol (values for the entire 5-day training period: *p* = 0.21, F(4,1) = 2.20; values for days 4 and 5 only: *p* = 0.038, F(1,1) = 273.3, *n* = 9 WT, *n* = 9 TG). Female PDE4D5-D556A transgenic mice performed differently from their male counterparts in this assay, in that they performed better initially but then seemed to learn at a slower rate overall, so that their overall performance was undistinguishable statistically from wild-type females (Figure 4b, values for the entire 5-day training period: *p* = 0.87, F(4,1) = 0.02, values for days 4 and 5 only: *p* = 0.29, F(1,1) = 7.11, *n* = 8 WT, *n* = 10 TG). However, when the data from each female genotype were normalized relative to its own day 1 performance, a statistically significant difference was seen on subsequent days (*p* = 0.0196, F(4,1) = 14.20). When data for sexes were pooled, the differences on days 4 and 5 were more discernable (Figure 4c, values for the entire 5-day training period: *p* = 0.42, F(9,1) = 0.71, values for days 4 and 5 only: *p* = 0.0073, F(3,1) = 42.56, *n* = 20 WT, *n* = 20 TG). In this assay, male mice swam somewhat more slowly than females, but there was no discernable difference in the swimming distance between wild-type and PDE4D5-D556A transgenic mice (i.e., distance traveled to reach the target, TG males, 18.93 ± 1.13; WT males, 19.84 ± 1.30, TG females, 20.59 ± 0.90; WT females, 20.12 ± 1.20; all means ± SEM, *p* = NS). Testing the performance of the mice to remember the correct quadrant, after 5 days of testing and upon removal of the target platform, showed significant variability, with no statistical significance seen (probe trials, Figure 4d, comparing quadrant A to quadrant R, sexes pooled, *p* = 0.087, F = (1,31)3.11, *n* = 16 TG, *n* = 17 WT).

## 3. Discussion

We developed transgenic mice that express a dominant-negative mutant (D556A) of a single PDE4 isoform, specifically PDE4D5, preferentially in the hippocampus and forebrain and shown that the transgene produces significant effects on behavior. The use of a dominant-negative transgene in this study, and in our prior study of PDE4B1 [133], is a novel approach to determining the functions of PDE4 isoforms in the CNS. Previously, studies of PDE4 function employed gene knockouts [27,137,138,139,140,141,142,143], lentiviral siRNA [142,144,145,146,147], or dominant-negative mutants encoded by adenoviruses [135,136]. We developed this approach after we and our collaborators carefully studied the effects of the PDE4D5-D566A mutant in cell-based assays [103,108,109], as described in more detail below. Based on the cell-based studies, and our prior experience with PDE4B1 [133], we felt that this mutant, when expressed as a transgene, would have a more isoform-selective effect than that seen in prior studies of *Pde4d* gene knockouts. An important reason for this prediction is that the mouse *Pde4d* and human *PDE4D* gene both encode 11 isoforms (Figure 1a; [73,88,96,97,98,99,100]), each with a distinct amino acid sequence and distribution in tissues. For this reason, *Pde4d*-/- mice have a phenotype that reflects the combined deficiency of all 11 PDE4D isoforms, which prevents analysis of the effect(s) of any individual isoform, such as PDE4D5. For this reason, our approach is likely to have greater selectivity for the PDE4D5 isoform than *Pde4d-/-* gene knockouts or siRNA. Our dominant-negative approach also follows the strategy used by several groups to study PKA and CREB in the CNS, in that transgenic mice expressing a dominant-negative PKA RIα subunit [177], or a dominant-negative CREB mutant [32,66,169,170,171,178,179,180], all have defects in various components of learning and memory.

We emphasize PDE4D5 for several reasons. First, human PDE4D5 (GenBank AF012073) and mouse PDE4D5 (GenBank XP_006517707.1) are extremely similar, being identical in length and having a 98% amino acid identity. Secondly, PDE4D5 mRNA is expressed in several regions of the mouse CNS (see background for details), compatible with it being targeted by a transgene driven by the αCaMKII promoter. Finally, PDE4D5 interacts with several proteins, most notably RACK1 and β-arrestin2, both of which appear to have a much higher selectivity and avidity for PDE4D5 than for any other PDE4 isoform (see background for references). In particular, PDE4D5 appears to have a pivotal role in regulating signaling through the β_2_-adrenergic receptor [108,109,111,112,148,149]. Therefore, study of PDE4D5 might provide insights into the role(s) of β_2_-adrenergic signaling in the CNS.

In the current study, the PDE4D5-D556A-encoding transgene was expressed off the αCaMKII promoter [155,156,157]. We also used this promoter in a recent prior study to express a dominant-negative mutant of PDE4B1 [133]. We chose this promoter as it is active preferentially in forebrain excitatory neurons, including those in the hippocampus, amygdala, cortex, and striatum [155,156]. This choice may also minimize any effects of the transgene on prenatal development, as the αCaMKII promoter becomes active several days after birth [181], when many CNS pathways are already formed [157]. This promoter has been employed in the development of knockout and transgenic mice used in many studies of learning and memory [155,157,169,170,171,177,182,183,184] and other neurobiological phenotypes [185]. However, given the differences in the regional expression of αCaMKII and PDE4D5, we do not expect that all of the biological functions of PDE4D5 are disrupted equally in all areas of the brain where it is normally expressed. Additionally, the expression of the transgene may vary according to its insertion site. Our PDE4D5-D556A-encoding transgene is likely to be expressed in brain areas lacking endogenous PDE4D5; however, since extensive study of PDE4D5-D556A in cell-based assays has shown that all of its biological effects are mediated through its action on endogenous PDE4D5, as described in the next three paragraphs, the transgene is unlikely to have a meaningful biological effect in those areas.

When overexpressed, the PDE4D5-D556A protein produces no change in the total PDE4 enzymatic activity in cell-based systems. Instead, it disrupts PDE4D5 cellular function in a dominant-negative fashion by producing an equilibrium displacement of endogenous PDE4D5 from its protein partner(s), most notably RACK1 and β-arrestin2 [103,108,109,111,132]. Although an “off-target” effect of the PDE4D5-D556A protein on other PDE4 isoforms, notably other PDE4D isoforms, cannot be excluded completely, we believe this is unlikely, as PDE4D5 interacts highly selectively with different protein partners than other PDE4D isoforms do (see background for details). Therefore, the expression of PDE4D5-D556A should block PDE4D5 function in specific sub-cellular compartments where PDE4D5 is active, increase cAMP levels, and ultimately activate PKA. Activation of PKA is likely to lead to increased phosphorylation of CREB, a major PKA substrate, in regions of the CNS where our transgene is expressed, including area CA1 of the hippocampus and the dentate gyrus. Some effects of the transgene may also be mediated by other cAMP effectors, including EPAC [68,174,175] and/or cyclic nucleotide-gated ion channels [70]. This model of PDE4D5-D556A action does not require that global levels of cAMP change detectably with the PDE4D5-D556A-encoding transgene. Instead, tissue-level cAMP levels would reflect the cumulative effect of numerous elements in cAMP signaling, including the effects (potentially compensatory) of other PDE4 isoforms, as well as the effects of members of other PDE families. Indeed, it is conceivable, although not likely, that PDE4D5-D556A could lower cAMP levels in certain contexts, e.g., by shifting endogenous PDE4D5 into a subcellular compartment where it is not normally present.

It is possible that some of the effects of PDE4D5-D556A are mediated by its ability to interfere with the homodimerization of PDE4D5 [107,127,128,129,130,131]. This is because PDE4D5 interacts with β-arrestin2 only when PDE4D5 is monomeric [107]. Therefore, PDE4D5-D556A can act as a dominant-negative in two ways: (1) It can compete, as a monomer, with monomeric wild-type PDE4D5 for interaction with β-arrestin2, by binding directly to β-arrestin2; or (2) It can dimerize with wild-type PDE4D5; the resulting dimer does not interact with β-arrestin2. We did not detect any “off-target” effects of PDE4D5-D556A in these studies, as heterodimerization of PDE4 isoforms has not been observed [107,127], although it cannot be definitely excluded in the studies that have been performed to date.

Some of the effects of PDE4D5-D556A may be mediated through its effects on RACK1 and β-arrestin2, or other proteins that interact selectively with PDE4D5. By dramatically increasing scaffold sites for β-arrestin2 and RACK1, PDE4D5-D556A may sequester these proteins and thereby block their normal functions in cells. This effect would produce a CNS phenotype that would differ from that seen with pharmacological inhibition of PDE4D5, and especially from that seen with small-molecule inhibitors of the catalytic activity of PDE4D5. Such a mechanism might explain some of the behavioral effects that we see in our transgenic mice, as explained in more detail in the next paragraph.

Our PDE4D5-D556A transgenic mice have a phenotype that seems to affect hippocampal-dependent positional learning (place preference), based on their performance in the Morris water maze [176]. This result is consistent with prior studies that show that cAMP signaling mediated by CREB, and/or possibly EPAC, plays an essential role in learning and memory (see [64,65,66,68,69] for reviews). However, the direction and magnitude of the effect that we observed were intriguing. Given that PDE4 inhibitors are being developed as enhancers of cognition and memory, we were expecting that attenuation of PDE4 activity in the brain would produce an enhancement of performance in tests of learning and memory. However, our PDE4D5-D556A mutant appeared to produce a deleterious effect in the Morris water maze. Although the mechanism(s) of this paradoxical effect are uncertain, it is possible that it reflects the ability of PDE4D5-D556A to sequester RACK1 and/or β-arrestin2, as described above. Although further experiments are necessary to support this hypothesis, our data clearly support a role for PDE4D5 and its specific partner proteins in hippocampal-dependent learning and memory.

In our studies, the PDE4D5-D556A transgene had no detectable effect on associative fear conditioning. Fear conditioning, especially contextual fear conditioning, was tested because it is an amygdala-dependent memory test mediated, at least in part, by CREB action in the hippocampus [65,66,168,169,170,171,172]. One potential reason for our results is the notably high baseline performance of wild-type C57BL/6J mice in this assay, which meant that any improvement in the transgenic mice might have been obscured, especially when strong stimuli were used in conditioning, as we did here. A weak effect in this assay might also have been missed because of the sample size; however, sample sizes were limited by animal ethics issues and financial resources. Finally, our PDE4D5-D556A transgene may not have been expressed at sufficiently high levels in the amygdala to suppress the action of endogenous PDE4D5 in this area.

Given their phenotype in the open-field test, our PDE4D5-D556A transgenic mice appear to have a behavioral phenotype that affects activity. This effect was clearly sex dependent, in that transgenic females were less active than wild-type females, while transgenic males had a tendency to be more active than wild-type males. Overall, however, the PDE4D5-D556A transgene had a weaker effect in males than the PDE4B1-D564A transgene, which produced a highly significant increase in the activity of males in the open-field test [133]. The potential neurophysiological mechanism(s) and/or anatomical site(s) of action for these sex differences is not certain; it is possible that there are sex-dependent differences in the expression of the transgene, although this has not been reported previously for transgenes expressed off the αCaMKII promoter [155,156,157]. Nonetheless, these data demonstrate clearly that PDE4B1 and PDE4D5 have significantly different functional roles in the CNS and that our dominant-negative approach clearly has sufficient isoform selectivity to distinguish between these differing functional roles. As PDE4D5-D556A transgenic mice had behavior indistinguishable from wild-type mice in the elevated plus maze, we also conclude that the PDE4D5-D556A transgene does not appear to have an anxiogenic effect.

Two different groups have studied the behavioral phenotypes of *Pde4d-/-* mice [27,137,142]. Some of these studies show that *Pde4d-/-* mice have behavior that mimics the effects of some antidepressants [137], for example, decreased immobility in forced-swim and tail-suspension tests. One group has concluded that *Pde4d-/-* mice do better in tests of learning and memory [142], while another group, studying an apparently identical genotype, did not detect this effect [27]. Comparing these data to those in the present study is complicated further by the fact that *Pde4d-/-* mice appear to have phenotypes that are affected by processes in areas of the brain, notably the striatum, beyond those in the forebrain/hippocampus, which was the focus of the present study. Finally, all *Pde4* knockouts have significant non-CNS phenotypes [186,187], such as slow growth, small adult size, and impaired fertility. The differences between the findings in the present study and those obtained by these two groups with *Pde4d-/-* mice probably also reflect isoform differences (PDE4D5 vs. all PDE4D isoforms). Differences in assay conditions, genetic background, age at the time of study, or other factors could also be responsible.

In humans, a specific set of mutations in *PDE4D* cause acrodysostosis, a disorder that affects bone formation [188,189,190,191,192]. Patients with acrodysostosis and *PDE4D* mutations also have significant intellectual disability. Acrodysostosis mutations cluster in a specific region of the PDE4D protein that is essential for its dimerization and regulation by phosphorylation by PKA [127]. Unlike our PDE4D5-D556A mutant, acrodysostosis mutations do not affect amino acids that are directly involved in catalysis [127]. The CNS deficiencies that are seen in acrodysostosis appear to be considerably more severe than those seen in *Pde4d-/-* or PDE4D5-D556A transgenic mice, affecting multiple aspects of cognition and memory. It is uncertain whether this reflects a purely species difference or a different mutation mechanism (unlike our dominant-negative PDE4D5-D556A mutant, the *PDE4D* acrodysostosis mutants appear to have a “gain-of-function” effect, in that they render the enzyme constitutively insensitive to PKA regulation). Although much remains to be learned about the CNS phenotypes of *PDE4D*-mutant acrodysostosis, the available data demonstrate the profound effects of PDE4D alterations in the CNS and thereby the essential role of PDE4D in CNS function in humans.

Our data, along with the observations from other groups summarized above, show convincingly that PDE4D5 has an important role in the CNS. Therefore, our results, and those of other groups, provide strong justification for the focusing on PDE4D, and PDE4D5 in particular, as a target for drug development [38,43,45,46,47,48,58,60,193,194,195]. Recent human trials of pan-selective PDE4 inhibitors active in the human CNS have shown some promise [9,10,12]. To date, the development of PDE4-selective inhibitors in many CNS indications has been limited by nausea and emesis [86]. Many of the emetogenic effects of PDE4 inhibitors are mediated by the area postrema, where several PDE4B and PDE4D isoforms are known to be expressed [82,196]. However, it should still be possible to target PDE4 informs or conformers that are not present in the area postrema, with potential value in the treatment of depression and disorders of learning and memory. Novel approaches to targeting these isoforms might include CRISPR or shRNA specific to each individual isoform, or small-molecule disruptors of the interaction of specific isoforms with their specific binding partner proteins.

## 4. Materials and Methods

### 4.1. Generation of Transgenic Mice

Transgenic mice expressing PDE4D5-D556A, under the control of the α-calmodulin kinase II (αCaMKII) promoter, were generated using methods that we described previously for dominant-negative PDE4B1 [133]. The PDE4D5-D556A mutant (Asp556Ala, GAC to GCC) in full-length human PDE4D5 (Figure 1c, GenBank AF012073, [88]), with sequences encoding an 11-amino-acid carboxyl-terminal VSV epitope [197], was generated by site-directed mutagenesis and cloned into the expression plasmid pMM403 ([155,156]; generously provided by Mark Mayford, Scripps Institute, La Jolla, CA, USA). All animal work was performed under protocols approved prior to the initiation of the study (Animal Project Number: 08010842) by the Institutional Animal Care and Use Committee (IACUC) of the University of Alabama at Birmingham and followed the NIH guide for the care and use of laboratory animals and other national regulations and policies. All efforts were made to minimize suffering and no surgery was performed.

### 4.2. Immunoblotting

Monkey COS-7 cells (purchased from ATCC, Manassas, VA, USA) were transfected with the plasmid pcDNAN79D556AVSV, or with vector pcDNA3 (Invitrogen, ThermoFisher, Carlsbad, CA, USA), using technology that we described previously [198]. The plasmid pcDNAN79D556AVSV encodes human PDE4D5-D556A, with the carboxyl-terminal VSV epitope [197]. Extracts from COS-7 cells and brain, respectively, were prepared using techniques we described previously [133,198,199]. The extracts were subjected to LDS-PAGE (Novex, Invitrogen, ThermoFisher, USA) and immunoblotted. For comparing apparent molecular weights under denaturing conditions, samples were run in parallel lanes on the same gel and then transferred to a single filter for immunoblotting. The filter was then cut in half cross-wise, with the top half incubated with an antibody to VSV (mouse, clone P5D4, Millipore-Sigma, St. Louis, MO, USA, ref. [197], 1:5000) in Tris-buffered saline (TBS) with 0.1% Tween-20 and 0.5% non-fat dry milk for 1 h. As a loading control, the bottom half was incubated with an antibody to GAPDH (rabbit, clone D16H11, Cell Signaling Technologies [CST], Danvers, MA, USA, 1:2000), as for VSV, except that incubation was in Tris-buffered saline (TBS) with 0.1% Tween-20 and 5% non-fat dry milk for 24 h at 4 °C. Primary antibody incubations were then followed by 2 washes in TBS with 0.1% Tween-20. Secondary antibody incubations (mouse, SC-516102, Santa Cruz Biotechnology, Santa Cruz, CA, USA, 1:5000; rabbit, CST 7074, 1:5000) were performed in the same buffer, followed by 3 washes in the same buffer. Signals were developed with ECL (Pierce, ThermoFisher, USA). Imaging was performed with a C-DIGIT scanner and ImageStudio software, version 5.2 (LI-COR Biosciences, Lincoln, NE, USA).

### 4.3. Behavioral Assays

Mice were tested at 8–12 weeks of age. Mice were housed in cages containing 2 to 5 animals on a 12-h light:12-h dark cycle, with all behavioral testing performed in the light phase. The experimenter was blind to the genotype of the mice during testing and data collection/analysis. Testing for the basic neurobiology (SHIRPA) battery was performed on cohort 1 (2 generations backcrossed into C57BL/6J). Open-field testing, followed about 7 days later by fear conditioning and then acoustic startle, and then followed about 7 days later by the elevated plus maze and the Morris water maze, were performed on a separate cohort (cohort 2; 3 generations back-crossed into C57BL/6J). Testing in the Morris water maze was then performed on a third cohort (cohort 3; 4 generations backcrossed into C57BL/6J) and the results pooled with those from cohort 2. For each experiment, sample sizes were determined after review of the previously published descriptions of each procedure (for details, see the references cited in Section 4.4, Section 4.5, Section 4.6, Section 4.7, Section 4.8 and Section 4.9). Males and females were tested throughout and results (WT vs. TG) were analyzed separately for each sex. In addition, the results for both sexes were pooled, and statistical tests performed on this pooled sample set. For all behavioral assays, data were collected in Excel 2013 (Microsoft, Redmond, WA, USA) and statistical analysis and graphs prepared with SigmaPlot 12.0 (Systat, San Jose, CA, USA).

### 4.4. Basic Neurobiological Battery

This was performed according to the SHIRPA protocol [158,159], as we described previously [133].

### 4.5. Open-Field Testing

This was performed using a photobeam apparatus [167,200,201], as we described previously [133].

### 4.6. Acoustic Startle and Prepulse Inhibition

This was performed using a dedicated apparatus [167,202], as we described previously [133].

### 4.7. Elevated Plus Maze

This test, a standard test for measuring anxiety-like behavior [203], was performed as we described previously [133].

### 4.8. Context-Dependent and Cue-Dependent Fear Conditioning

This was performed as described previously by numerous groups [167,200,201,204,205,206,207] and exactly as reported previously by us [133].

### 4.9. Morris Water Maze

The Morris water maze test [176] measures the ability of an animal to recall the position of a hidden platform (i.e., the escape platform) upon training. Therefore, responses were measured after a series of training steps. Mice were trained in a 120-cm blue circular pool containing clear water and a 10-cm round escape platform located 0.5 cm below the water surface. Water temperature was maintained at 20–22 °C. Mice were placed into the water facing the wall, in different quadrants on a random basis, and allowed to search for the platform for 60 s. If the mice did not locate the platform at the end of 60 s, they were guided to it. The mice were permitted to stay on the platform for 10 s and then were returned to their cages. All trials were performed at the same time of day (±1 h) during the animals’ light phase. Each mouse was given two blocks of 4 trials a day for 5 consecutive days, with a 20-min inter-trial interval. For probe trials (i.e., response assessment), which were performed on day 5, each mouse was placed in the pool in the absence of the escape platform and its search monitored for 60 s. The movements of the mice during training and in the probe trials were recorded by a continuous video tracking system (Ethovision, Noldus, The Netherlands) and analyzed using Hvswater. During training sessions, the time (i.e., escape latency) and distance for the mouse to locate the platform were recorded. During the probe trial, the time spent searching each quadrant (quadrant search time) was recorded. Escape latency and distance data were analyzed with a two-way (genotype x trial block) ANOVA with repeated measures. Search time was analyzed with a one-way ANOVA.

### 4.10. Availability of Data and Materials

The datasets used and/or analysed during the current study are available from the corresponding author on reasonable request.
